# Single-Particle Catalysis: Revealing Intraparticle Pacemakers in Catalytic H_2_ Oxidation on Rh

**DOI:** 10.1021/acscatal.1c02384

**Published:** 2021-07-27

**Authors:** Johannes Zeininger, Yuri Suchorski, Maximilian Raab, Sebastian Buhr, Henrik Grönbeck, Günther Rupprechter

**Affiliations:** † Institute of Materials Chemistry, TU Wien, Getreidemarkt 9, Vienna 1060, Austria; ‡ Department of Applied Physics and Competence Centre for Catalysis, Chalmers University of Technology, Göteborg 41296, Sweden

**Keywords:** nanoscale system, surface reaction, single-particle imaging, chemical oscillations, intraparticle pacemaker, atomic arrangement, interfacet communication

## Abstract

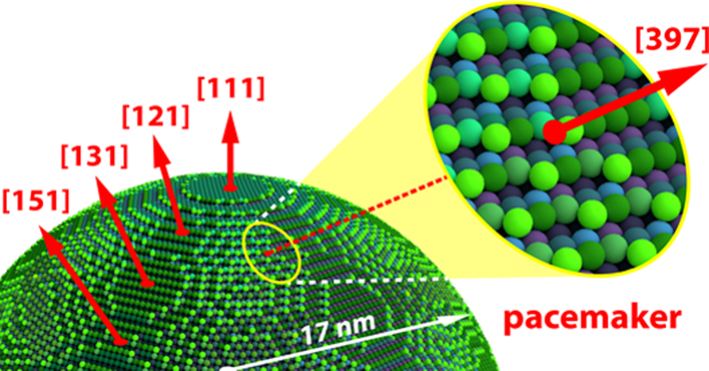

Self-sustained oscillations in H_2_ oxidation on a Rh nanotip mimicking a single catalytic nanoparticle were studied by *in situ* field emission microscopy (FEM). The observed spatio-temporal oscillations result from the coupling of subsurface oxide formation/depletion with reaction front propagation. An original sophisticated method for tracking kinetic transition points allowed the identification of local pacemakers, initiating kinetic transitions and the nucleation of reaction fronts, with much higher temporal resolution than conventional processing of FEM video files provides. The pacemakers turned out to be specific surface atomic configurations at the border between strongly corrugated Rh{973} regions and adjacent relatively flat terraces. These structural ensembles are crucial for reactivity: while the corrugated region allows sufficient oxygen incorporation under the Rh surface, the flat terrace provides sufficient hydrogen supply required for the kinetic transition, highlighting the importance of interfacet communication. The experimental observations are complemented by mean-field microkinetic modeling. The insights into the initiation and propagation of kinetic transitions on a single catalytic nanoparticle demonstrate how *in situ* monitoring of an ongoing reaction on individual nanofacets can single out active configurations, especially when combined with atomically resolving the nanoparticle surface by field ion microscopy (FIM).

## Introduction

Nanosized metal particles, which are the “workhorses” of catalysts, can nowadays be prepared with high precision with respect to their size, shape, and chemical composition.^1−3^ However, a certain size distribution and structural heterogeneity are unavoidable. Therefore, studies of structure–performance relationships would strongly benefit from examining catalytic reactions on single nanoparticles. In the last years, significant progress has been made in developing novel approaches for single-particle catalysis: single-molecule fluorescence microscopy, surface-enhanced Raman spectroscopy, nanoinfrared spectroscopy, nanoplasmonic sensing, X-ray microscopy, and others.^4−6^ In addition, remarkable advances have been made in resolving the atomic-scale structure of nanoparticles in gas and liquid phases.^7−9^

Nevertheless, there is still need for real-time methods for monitoring catalytic reactions with intraparticle lateral resolution, that is, observing differences in activity at various surface sites of a single particle, in parallel to collecting information on the particle structure. In this respect, the apex of a sharp metal nanotip can be employed as a model nanoparticle, since it exhibits the main property of a nanoparticle, namely, the presence of differently oriented nanofacets on its surface. In contrast to a true nanoparticle, such a nanotip can be characterized with atomic resolution by field ion microscopy (FIM), providing a well-defined model system, and its adsorption and reaction properties can be *in situ* monitored by FIM and by field emission microscopy (FEM). As for any model, there are differences between the model and modeled system; the size of the tip apex is somewhat bigger than that of typical nanoparticles, and the tip is not supported by an oxide. Nevertheless, nm-scale phenomena with closely interwoven temporal and spatial components, for example, catalytic ignition or self-sustaining oscillations, are accessible with these techniques. Already in 1993, the first *in situ* visualization of the oscillating CO oxidation on a Pt nanotip by FEM and FIM was reported.^10,11^ In addition to this model reaction on platinum group metals,^12−15^ FEM/FIM was also applied to a few other oscillating reactions, such as NO reduction^16,17^ and H_2_ oxidation.^18−20^

**Figure 1:**
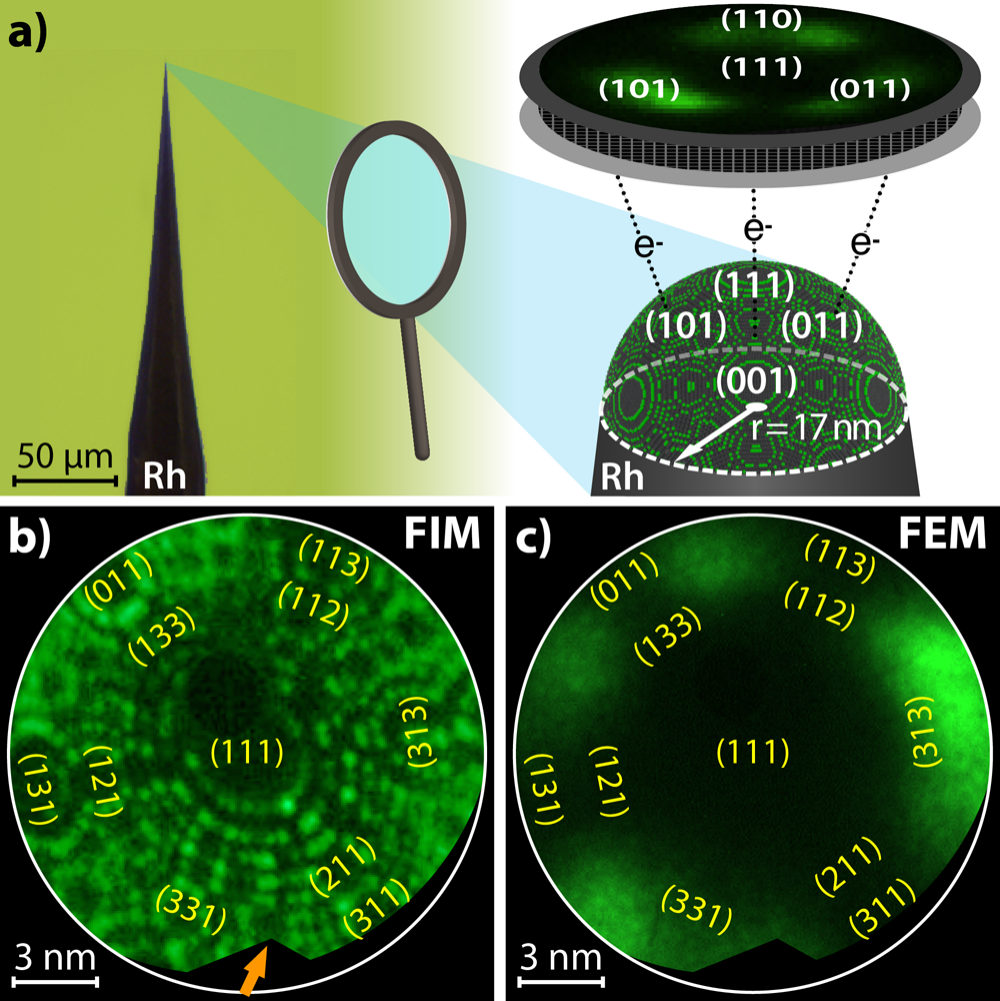
Apex of a Rh nanotip as model of a catalytic nanoparticle: (a) optical image of the Rh nanotip and a ball model of its hemispherical apex (*r* = 17 nm). The image on the screen is a point projection of the sample surface formed by field-emitted ions (FIM) or electrons (FEM); (b) atomically resolved FIM image of the Rh nanotip, obtained using Ne ^+^ ions (*T* = 77 K). Facets representing the main crystallographic orientations are indicated. The black spot above the (111) facet is the probe hole. The orange arrow marks a defect on the edge of the channel plate; (c) Same Rh tip imaged by FEM.

In both FEM and FIM, the image is generated by field-induced tunneling of electrons: in FEM, the electrons tunneling from the imaged surface sites into vacuum directly form the image on the screen (Figure 1); in FIM, electrons tunnel into the sample surface from the imaging species (gas atoms or molecules), leaving behind ions, which accelerate toward the screen and create the image. The image contrast in FEM is formed by the local variations in the work function, convoluted with the local field contribution,^21^ whereas the local field-dependent probability of field ionization creates the contrast in FIM.^22^ This fundamental difference in the image formation governs possible field effects on the imaged processes. The field-induced redistribution of electron density near the positively charged specimen in FIM^23^ may modify the binding energy of adsorbates,^24^ thus influencing surface reactions.^25^ In contrast, such an electron density redistribution cannot take place in FEM, since the electrons would emit from the negatively charged specimen way before the field strength necessary for field-induced changes is achieved. Therefore, in FEM, no field effects on the binding energy of reactants such as O, CO, or H can occur, and the corresponding catalytic reactions, such as CO or H_2_ oxidation, remain unaffected. This was directly proven by applying a pulsed field of varying duty pulse cycles.^25^ Apart from this essential advantage, the FEM image formation mechanism leads, due to exponential dependence of the electron emission yield on the local work function, to a huge dynamic range of the signal, which may exceed the capabilities of an image intensifier. The intensity from “bright” regions of the image may run into “clipping”, while the less-emitting “dark” regions remain featureless (Figure 1c). When fast surface processes are visualized, such as diffusion or reaction front propagation, the low image intensity limits the recording rate, hindering the acquisition of visual information. Clearly, an unrestricted acquisition of the local intensity signal in the entire field of view is highly desired, including the seemingly “invisible” regions. Even more so, the recently developed *kinetics by imaging*(26) approach could then also be applied to sample regions that were previously just “blind spots”. Such an approach is particularly important in the case of nanotips since due to the nm size of the catalytically active surface and high-vacuum conditions, the number of product molecules is hardly measurable by mass spectroscopy. In the present contribution, we apply a novel FEM image-processing method, allowing us to track “invisible” nanosized reaction fronts, and the proper orthogonal decomposition (POD) method to reveal the spatial synchronization of self-sustained oscillations in catalytic hydrogen oxidation on a rhodium nanotip, which models a single catalytic nanoparticle. Following this new approach, unprecedented insights into the origin and role of intraparticle pacemakers, which initiate kinetic transitions and the nucleation of reaction fronts, have been obtained. In order to concentrate on spatially synchronized effects, a nanotip with an apex radius of solely 17 nm was deliberately chosen to avoid the collapse of diffusional coupling occurring on nanotips with a significantly larger radius.^27^

## Experimental Section

For the present experiments, the apex surface of a Rh nanotip (Figure 1a) was shaped and cleaned by field evaporation at 77 K. Subsequently, the sample was characterized with atomic resolution by FIM (Figure 1b), and crystallographic orientations of nanofacets were deduced by comparison of the FIM image with the stereographic projection of the corresponding fcc lattice. The atomically clean Rh nanotip was then imaged by FEM (Figure 1c). The FEM/FIM chamber was used as a flow reactor for catalytic H_2_ oxidation on Rh at constant partial pressures of *p*_H_2__ = 2.3 × 10^–6^ mbar and *p*_O_2__ = 1.6 × 10^–6^ mbar and at constant temperatures of 430, 440, and 450 K. More experimental details are presented in Supporting Information S1.

## Results

**Figure 2:**
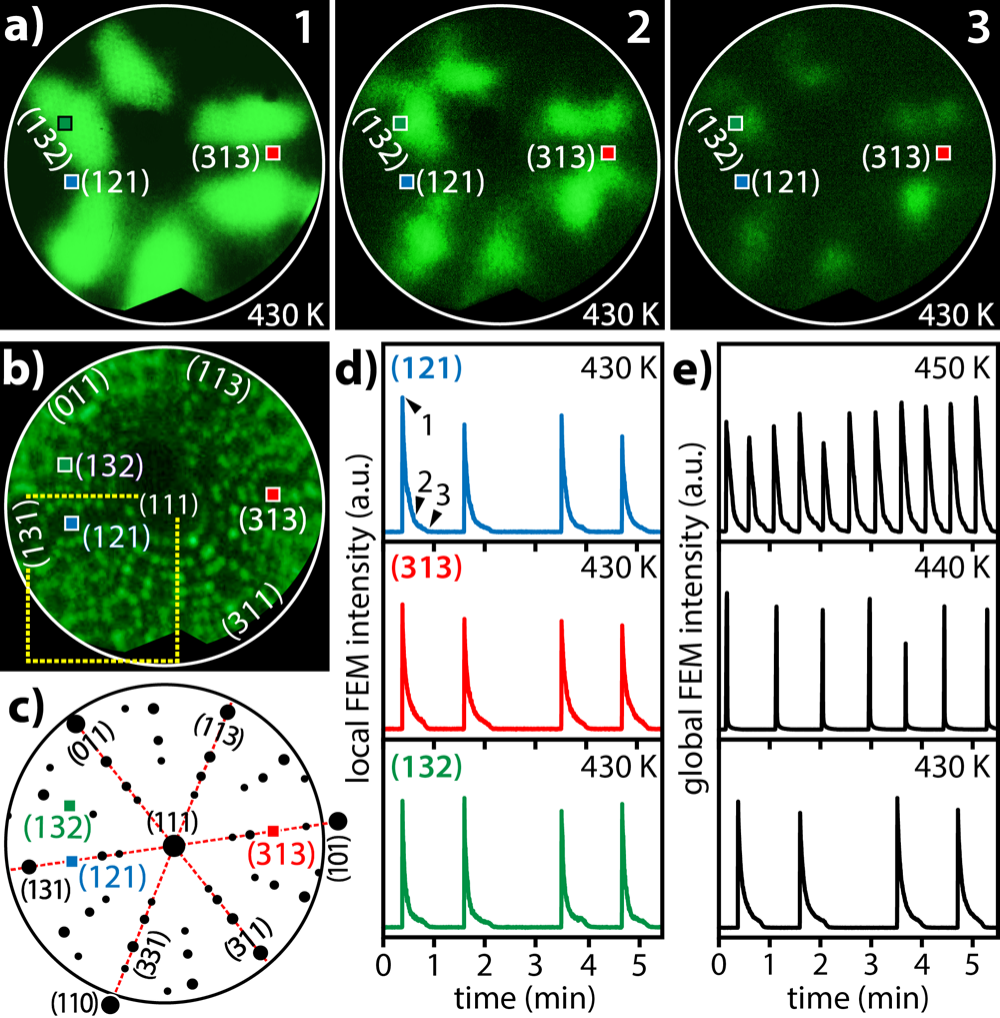
Catalytic H_2_ oxidation on a (111)-oriented Rh nanotip at *p*_H2_ = 2.3 × 10^–6^ mbar and *p*_O2_ = 1.6 × 10^–6^ mbar and at constant temperatures of 430, 440, and 450 K: (a) *in situ* FEM video frames acquired at 430 K. The corresponding time points 1–3 are marked in panel (d). The green, blue, and red squares indicate ROIs placed on the (132), (121), and (313) facets, respectively; (b) Ne^+^ FIM image of the clean Rh-nanotip surface. The same ROIs as in panel (a) are indicated. The outlined yellow rectangle represents the region discussed in Figure 4a; (c) crystallographic map corresponding to the field of view in panel (b); (d) local FEM intensity registered in ROIs marked in panels (a–c) at 430 K; (e) examples of global FEM intensities at different temperatures.

Under the applied conditions, the reaction exhibits self-sustained oscillations, alternating between the catalytically inactive and active states, similar to field-induced oscillations in H_2_ oxidation on Rh nanotips previously observed by FIM.^18,19^ In the present FEM studies, oscillations are induced by field effect-free formation/depletion of subsurface oxygen, as proven by PEEM/SPEM studies on planar Rh samples.^28−30^ Since the inactive (oxygen-covered) and active (low oxygen and hydrogen coverage) surfaces significantly differ in their work functions and thus in the FEM image brightness, the switching between the active and inactive state can be monitored *in situ* by FEM.^31^ While the inactive (oxygen-covered) Rh surface exhibits dark contrast (high work function), the active surface with low oxygen and hydrogen coverage appears bright (low work function). By placing regions of interest (ROIs) at chosen positions, as exemplarily shown in Figure 2a, this process can also be traced locally for crystallographically different nanofacets present on the tip surface. Figure 2d,e shows the results of the analysis of the real-time video files recorded during the ongoing oscillating reaction, contrasting local (Figure 2d) FEM intensities of the ROIs marked in Figure 2a and global FEM intensities marked in Figure 2e (integrated over the entire field of view). The resulting oscillations appear as sharp “blinking” (see the original FEM recording at 430 K in Movie S1).

Apart from the sharp blinking, the observed oscillations are characterized by their apparent coherence over the majority of the sample surface, as is clearly visible in Figure 2d comparing the oscillations in three different ROIs. This impression, resulting from both visual inspection of video files and from local intensity analysis within particular ROIs, requires, however, a quantitative proof.

Particularly, the claimed “majority of the surface” needs to be evaluated and quantified. To obtain a clear assessment of the degree of coherence, we applied POD, also known as Karhunen–Loève (KL) decomposition, to the FEM video data. This method was already proven effective in the detection of coherent spatio-temporal modes, for example, in hydrodynamics^32^ or in spatio-temporal patterns in catalysis.^33^ Later on, we have used POD for the analysis of reaction-induced fluctuations^34^ and spatially coupled ignition in CO oxidation.^35^

The POD application to the present FEM video data is based on the fact that any spatio-temporal signal *W*(***x***,*t*), with ***x*** as the position vector, can be decomposed into time-independent spatial coefficients (POD modes) *b*_*n*_(***x***), which form the KL-basis, and their time-dependent coefficients *a*_*n*_(*t*), from which the original signal can be reconstructed
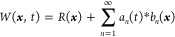
1

In the case of discrete video data, *W*(***x***,*t*) = *W*(***x***_***i***_,*t*_*j*_), with *i* being the number of the specific pixels and *j* being the video-frame number, and *R*(***x***) being a constant image, consisting of pixel intensities individually averaged over the entire time period. The details of the POD analysis are presented in Supporting Information S2.

**Figure 3:**
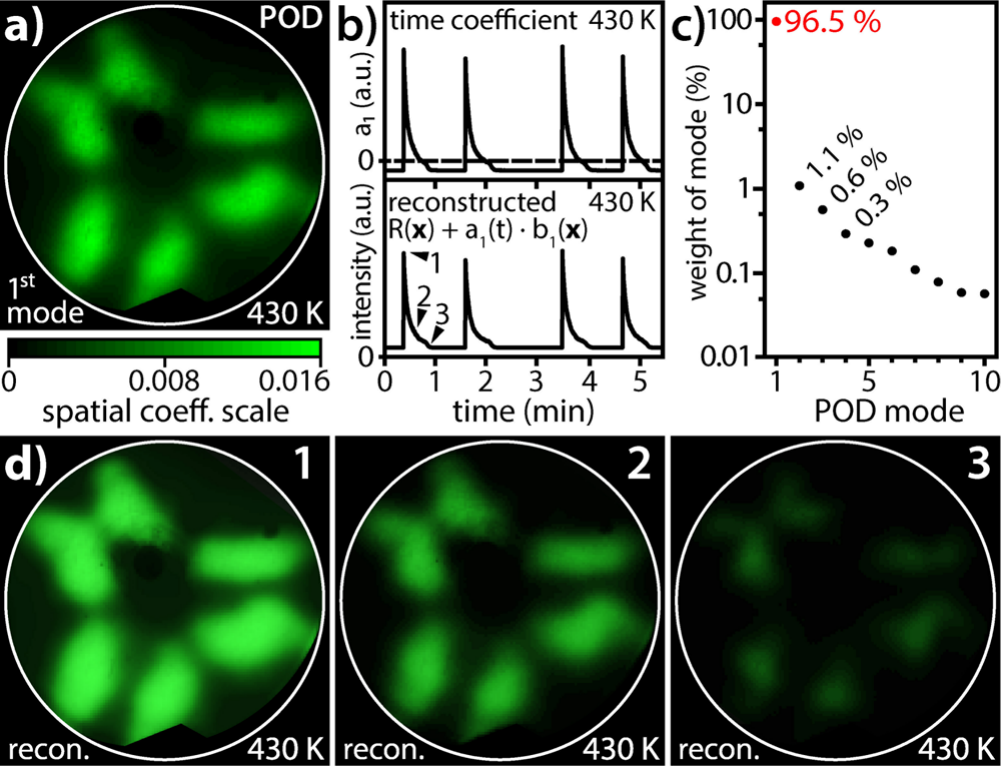
POD analysis of oscillations at 430 K: (a) first POD mode capturing the FEM field of view. The color code bar is shown below; (b) top: time coefficient *a*_1_ of the first POD mode; bottom: reconstructed global FEM intensity; (c) relative weight of the first 10 POD modes (logarithmic scale); (d) FEM video frames at time points indicated in panel (b), that is, the same as in Figure 2a.

If the first few POD modes already represent most of the signal weight, that is, they properly capture the overall dynamics of the system, the dimensionality of the signal can be significantly reduced without losing important information. We used this possibility to obtain the main features of the present FEM video data set. Figure 3 shows the application of POD to the same FEM video sequence that was used above for the local FEM image intensity analysis (Figure 2).

The first POD mode *b*_1_(***x***) is displayed in Figure 3a, and the upper panel in Figure 3b shows the corresponding time coefficient *a*_1_(*t*). Since the first mode already captures most (96.5%) of the signal (Figure 3c), the POD reconstruction (eq 1) of the FEM video data using solely the first-mode contribution almost perfectly restores the integrated FEM intensity (bottom panel of Figure 3c). The entire spatio-temporal FEM video signal can be reconstructed in this way as well: *cf*. the original (Figure 2a) and reconstructed FEM video frames (Figure 3d) and the corresponding Movies S1 (original) and S2 (reconstruction).

Since the first POD mode consists of a constant spatial image oscillating with the time-dependent amplitude *a*_1_(*t*), this means that different regions of the tip surface (*i.e.*, differently oriented (hkl)-facets) oscillate in a coherent way. The higher POD modes hardly affect the overall dynamics of the system: already, the second mode contributes solely with 1.1%, with an exponential decrease in remaining contributions of higher modes. This suggests that contributions of higher modes are caused by fast local events such as reaction-induced fluctuations, which occur outside the bistability region in a spatially incoherent stochastic way.^34^ Such a stochastic fluctuating contribution causes also a certain irregularity in the periodicity of oscillations, as is visible in Figures 2 and 3.

**Figure 4:**
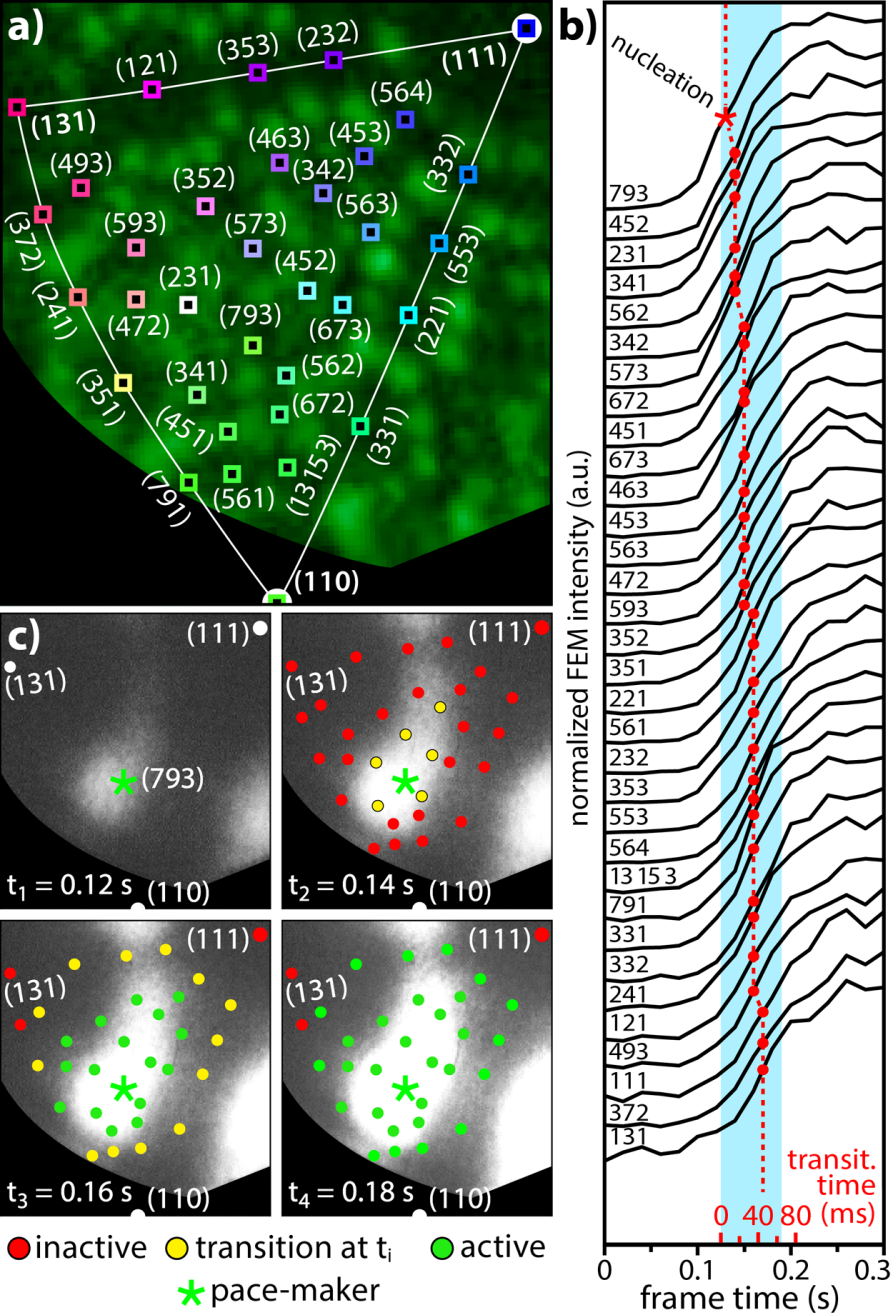
Spatial evolution of a kinetic transition during the oscillation half-cycle at *T* = 430 K: (a) segment of the FIM image with (hkl)-ROIs positioned on particular crystallographic nanofacets; (b) local FEM intensity curves registered in (hkl)-ROIs marked in panel (a) during a half-cycle of oscillations corresponding to the transition from the inactive to active states. Red points mark the inflection (transition) points, and the red star marks the pacemaker. The curves are sorted in the order in which the kinetic transition occurs; (c) four chosen *in situ* video frames (same region as in panel (a)) recorded at an interval of 0.02 s. The positions of “still inactive” (red), “just in transition” (yellow), and “already active” (green) are marked.

The repetitive kinetic transitions from the catalytically inactive to active states and vice versa, which form the observed oscillations, differ from the transitions occurring during isobaric ignition/extinction experiments^35,36^ solely by the origin of the trigger. In the present case, an internal feedback mechanism (formation/depletion of subsurface oxygen^28,29^) induces the transitions, instead of temperature variations in ignition/extinction experiments. In catalytic ignition, the transition point is defined as the inflection point in the time dependence of the reaction rate.^36^ This can also be applied to other types of kinetic transitions, including oscillations. The reaction rate in a Langmuir–Hinshelwood reaction follows, in a mean-field picture, the variations in the surface coverage of reactants, which also cause unambiguous variations in the FEM image intensity, which is why the latter reflects the local reaction rate. Although the exact shape of the time dependence of both curves (reaction rate and image intensity) may differ, the maxima, minima, and inflection points coincide, which is the basis for the *kinetics by imaging* approach.^26^ This is also valid for the local reaction rate down to the nanoscale, that is, each pixel of the FEM image carries the local kinetic information, and the points in time of local kinetic transitions during an oscillating cycle can be determined pixel-wise. Since kinetic transitions do not occur simultaneously over the sample surface, the spatial evolution of the transition points may provide information on how kinetic transitions travel over the surface. An example of such an evolution is presented in Figure 4: a segment of an atomically resolved FIM image, with particular Rh(hkl)-facets and ROIs placed at specific positions, is depicted in Figure 4a,b, displaying the time dependence of the local FEM intensity for every ROIs during the transition from the inactive to active state in a half-cycle of oscillation.

As results from the profiles in Figure 4b, all local transitions within the surface area shown in Figure 4a occur with a certain delay with respect to the Rh(793) facet that appears to be the transition pacemaker. This is illustrated in Figure 4c, in which the spatial distribution of the “still inactive” (red), “just in transition” (yellow), and “already active” (green) ROIs is marked. Since kinetic transitions in catalytic H_2_ oxidation on Rh are transported by propagating reaction fronts,^37^ the positions of the yellow points mark the current course of the reaction front. In principle, a reaction front is nothing else but a traveling continuum of transition points; thus, the evaluation shown in Figure 4 represents a novel method of tracking reaction fronts. It has to be noted that due to the relatively small local contrast differences and a high (on a nm scale) front velocity, the local propagation of fronts is neither visible to the naked eye nor can be detected with conventional image processing methods such as contrast stretching, histogram equalization, or convolution operations such as sharpening, noise reduction, embossing, and edge enhancement. The same holds true for the detection of the pacemakers: these are exclusively determined by the evaluation of the inflection points in the local intensity curves (Figure 4b).

The location where the inflection point appears first (easily detectable within the blue area) corresponds to the local pacemaker; in the present case, this is the Rh(793) facet. The recorded images, however, consist of 307,200 pixels, so that pixel-wise intensity analysis similar to that illustrated in Figure 4b would provide a fully resolved picture (pixel diagonal corresponds to ≈0.1 nm on the tip surface) of the front propagation. Therefore, a sophisticated automated transition point-tracking (TPT) procedure was developed that allows us to map the propagation of a reaction front with high spatial and temporal resolution. To achieve such a high temporal resolution, the intensity progression is reconstructed from the recorded frames by relying on the sigmoidal time dependency of the activity during the kinetic transition. Consequently, for each pixel, a logistic fit is applied to calculate the continuous function from the discrete local intensity values. Since TPT allows sub-frame rate positioning of the transition time points on the reconstructed curves, such a procedure “outsmarts” the acquisition speed of the charge-coupled device (CCD) camera (50 frames/s in the present case) and provides a much higher effective temporal resolution than conventional ROI evaluations. In a simplified view, TPT can be seen as a high-speed virtual readout of pixel intensities. More details of TPT processing are presented in Supporting Information S3.

**Figure 5:**
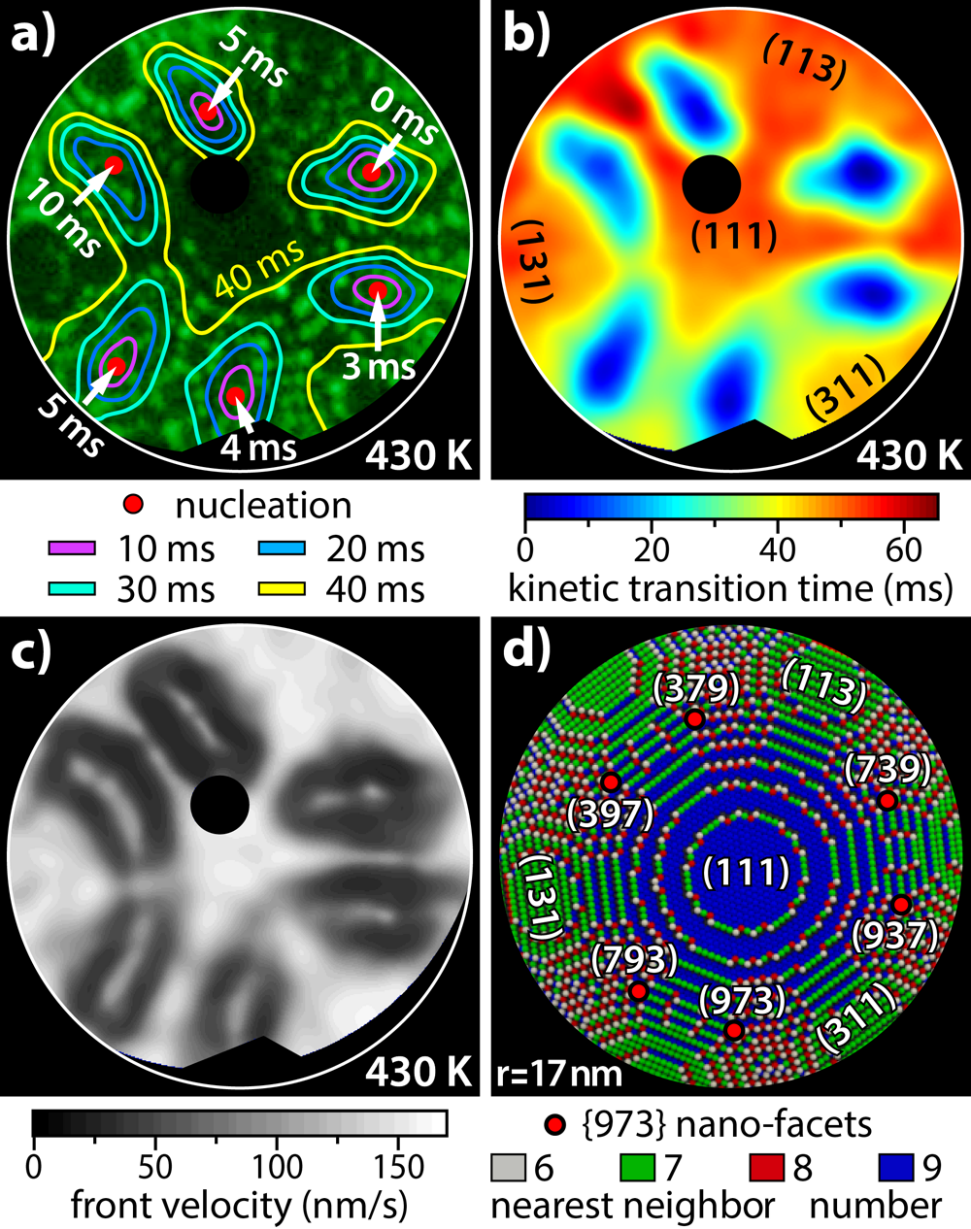
Pixel-by-pixel analysis of the spatial evolution of the kinetic transition from the inactive to active state during the oscillations in H_2_ oxidation on a Rh nanotip: (a) map of the positions of the oscillating reaction fronts superimposed on the FIM image. The time interval between the position lines is 10 ms, and red dots mark the pacemakers; (b) color-coded full “time scan” map describing the first 65 ms after the first front nucleation. The yellow contour represents the position of the reaction fronts 40 ms after front nucleation at Rh(739); (c) gray-scale coded velocity map (local front velocity at the time when the front passes the particular pixel); (d) ball model of the tip apex surface with color-coded local corrugation (represented by the number of nearest neighbors). The six local pacemakers (Rh{973} nanofacets) are indicated.

As an example of TPT analysis, the reaction front map illustrating the front nucleation centers and positions of the reaction fronts in the first 10, 20, 30, and 40 ms after the first front nucleation in the (739) region is shown in Figure 5a. The remaining five {973} pacemaker regions initiate the reaction fronts independently from each other within 10 ms. The corresponding reaction front map calculated with a time resolution of 1 ms can also be summarized in a “video”, illustrating the TPT-distilled local nucleation and propagation of reaction fronts (see Movie S3). Figure 5b displays a color-coded “time scan” transition map of the first 65 ms after the first front nucleation (corresponds to the time period highlighted in blue in Figure 4b), with each pixel colored according to its kinetic transition time.

The comprehensive collection of pixel-wise information about the momentary position of the reaction front allows us to calculate the local front velocities and to display them as velocity map. Figure 5c displays such a velocity map, that is, the gray-scale front velocity at the time when the front passes a particular pixel. Note that the velocity map shows an interesting correlation with the atomic surface corrugation map shown in Figure 5d: the regions with the highest velocity correspond to the lowest corrugations. This relation can be expected, but the precision of how even small deviations in atomic corrugation are reflected in the front velocity map is striking.

**Figure 6:**
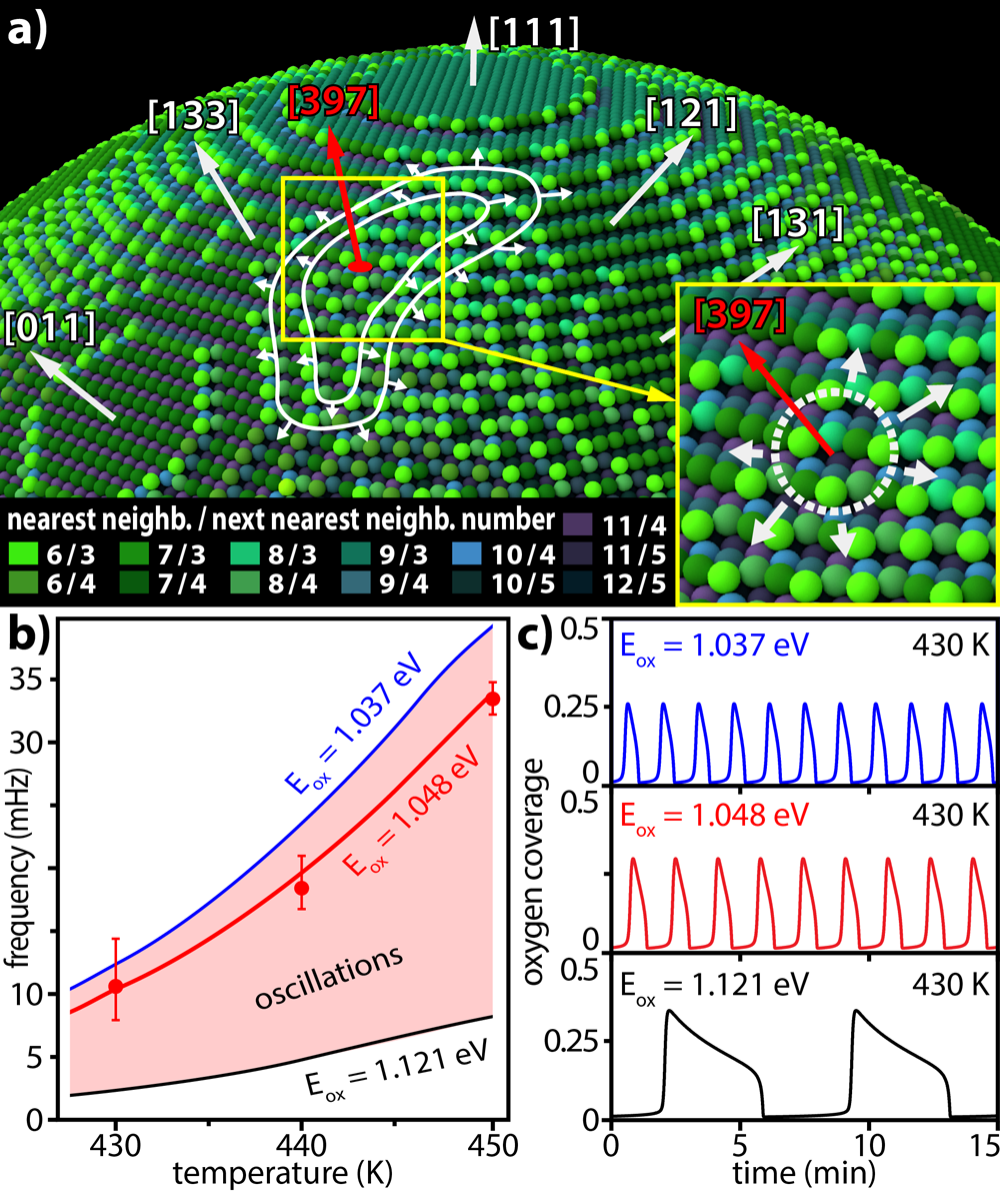
Reaction front propagation and the atomic surface structure: (a) ball model of the tip surface with color-coded local atomic corrugation represented by the number of nearest and next-nearest neighbors. The positions of the reaction front initiated in the (397) region (front nucleation at *t* = 10 ms) are marked at *t* = 20 ms and *t* = 30 ms. The inset shows the local crystallography of the pacemaker region; (b) microkinetic simulations of oscillations at *p*_O_2__ = 1.6 × 10^–6^ mbar and *p*_H_2__ = 2.3 × 10^–6^ mbar. Solid lines—calculations, red dots—experimental data; and (c) calculated oscillations of the oxygen coverage for different *E*_ox_ values.

The TPT analysis allows a detailed insight into the initial stages of the front propagation. In an early stage, reaction fronts initiated, for example, in the (397) region spread over the surface in an anisotropic way, as is schematically marked on the 3D ball model of the nanotip for the front positions at 10 and 20 ms after the local initiation (Figure 6a; note the color-coded coordination numbers). This behavior seems to be related to the particular atomic surface arrangement in the vicinity of the pacemaker. Recent studies of kinetic transitions in H_2_ oxidation on stepped Rh(hkl) surfaces revealed the unambiguous correlation between the “willingness” of a surface to undergo the kinetic transition and its “roughness”.^38^ The immanent correlation of the frequency in oscillating H_2_ oxidation and the surface structure of individual Rh(hkl) domains of a polycrystalline Rh foil confirms this observation.^29^ Thus, the particular surface structure of a Rh{973} nanofacet (see the inset in Figure 6a) seems to be the key in its pacemaker role in the present oscillations.

Our recent studies on planar stepped Rh surfaces identified atomically rough regions with a high kink and step density as being favored in subsurface oxygen formation;^28,29^ thus, one can expect that such regions may act as pacemakers. In fact, the observed pacemaker region Rh(397) is located at the edge between the “rough” region and the outer terraces of the Rh(133) facet. This provides a unique combination of the high local surface roughness with a “flat” zone in the form of an atomically narrow terrace. Such terraces might serve as channels for fast hydrogen diffusion, providing local excess of hydrogen necessary for the kinetic transition. This is supported by the observation that the reaction fronts preferentially expand along the terraces (Figure 6a and the velocity map in Figure 5c). Together, this indicates that in order to act as a pacemaker, a region which can form/deplete subsurface oxygen at a high rate also needs the atomic arrangement providing sufficient hydrogen supply to oxygen-poisoned areas to induce the nucleation of the reaction front.

To support the gained insights into the front nucleation process on the single-particle nanoscale, microkinetic calculations were performed, adapting the model previously used by McEwen et al. for field-induced oscillations.^19,39^ The adapted model focuses on the field effect-free formation/depletion of subsurface oxygen, acting as feedback of self-sustaining oscillations. More details on the microkinetic calculations under field effect-free conditions are presented in Supporting Information S4, and field effects in catalytic reactions are discussed in Supporting Information S5. The rate at which subsurface oxygen is formed essentially depends on the local activation energy of subsurface oxygen formation *E*_ox_, which is correlated with the local surface structure (atomic roughness) of the respective crystallographic area.^19,28^ Varying the *E*_ox_ value, the parameter region in the temperature/frequency space where the oscillations take place can be evaluated. Figure 6b shows such a region for the temperature range of 430–450 K where the oscillations were observed experimentally. When comparing the calculations with the experimentally observed oscillation frequencies, it appears that an *E*_ox_ of 1.048 eV simulates the pacemaker of the oscillations the best (red dots and line in Figure 6b). This is, however, not the lowest limit of *E*_ox_ for the calculated oscillations, which extends to 1.037 eV. The model yields kinetic oscillations only in a small range of *E*_ox_; this is consistent with the observed high sensitivity of the oscillations to the atomic structure.^28^ In accordance with the conclusions mentioned before resulting from the front propagation, this indicates that the ability to form subsurface oxygen is not the only deciding factor: flat terraces in the immediate neighborhood are necessary to stabilize the formation of a reaction front that spreads across the surface. The Rh{973} regions and their surroundings turned out to exhibit the optimal pacemaking properties under the applied experimental conditions.

### Summarizing Intrafacet and Interfacet Effects

Self-sustained oscillations in catalytic H_2_ oxidation on a Rh nanotip modeling a single Rh nanoparticle were observed using *in situ* FEM. Contrary to previous studies by FIM, the observed spatio-temporal oscillations were not generated by field-induced surface oxidation of Rh but instead result from coupling of the field-free subsurface oxide formation/depletion with reaction front propagation. A novel sophisticated method, TPT, applied to the FEM video recordings mimics the high-speed pixel readout of the CCD sensor and enables much higher temporal resolution than conventional video image processing. This allowed us to reveal how the local pacemakers initiate kinetic transitions and form reaction fronts in self-sustaining oscillations of H_2_ oxidation, that is, how the reaction proceeds over a single-nanoparticle surface. These most active sites turned out to be specific surface atomic configurations on the border between the strongly corrugated surface regions and adjacent relatively flat terraces. Such a specific combination provides, besides the corrugation-caused high permeability for oxygen incorporation under the Rh surface, also the required interfacet communication, that is, a sufficient hydrogen supply from the adjacent flat terraces necessary for the initiation of the kinetic transition. The present insights into the mechanism of kinetic transitions, occurring on a single catalytic nanoparticle, demonstrate the importance of *in situ* studies of kinetic processes on individual nanofacets, especially when combined with atomic resolution of the particle surface. The current approach is not limited to H_2_ oxidation or to oscillations but can also be applied to other catalytic systems where kinetic transitions take place.

## Supporting Information Available

More experimental details, explanation of POD, TPT, and microkinetic modeling, and explanation of the absence of field effects (PDF)
*In situ* FEM video (24 × speed) at 430 K, oscillations occurring during the hydrogen oxidation reaction on the apex of a Rh nanotip, and “blinking” character of the oscillations (MP4)
Video (430 K, 24× speed) reconstructed from the first POD mode capturing most of the signal (MP4)
TPT simulation of the reaction front propagation during the kinetic transition (0.01 × speed) with local front nucleation centers indicated (MP4)
