# Supplementary material for: Single-Particle Catalysis: Revealing Intraparticle Pacemakers in Catalytic H2 Oxidation on Rh
Source: ACS Catal. 2021 Jul 27;11(15):10020–7. doi: 10.1021/acscatal.1c02384 (PMC8353627; doi:10.1021/acscatal.1c02384)
Supplement: Supplementary file 1 — cs1c02384_si_001.pdf [file cs1c02384_si_001.pdf]

Supporting Information

Single particle catalysis: revealing intraparticle pacemakers in catalytic H_2 oxidation on Rh

Johannes Zeininger[1], Yuri Suchorski[1], Maximilian Raab[1], Sebastian Buhr[1], Henrik Grönbeck[2], Günther Rupprechter[1]*

[1]Institute of Materials Chemistry, TU Wien, Getreidemarkt 9, 1060 Vienna, Austria

[2]Department of Applied Physics and Competence Centre for Catalysis,

Chalmers University of Technology, 41296 Göteborg, Sweden

**Correspondence to:* guenther.rupprechter@tuwien.ac.at*

S1: Experimental Details

The experiments were performed in an all-metal field ion/electron microscopy (FIM/FEM) setup, containing a specimen holder, a channel-plate/screen assembly for imaging by ions of noble (Ne) or reactive (H_2, O_2) gases (in the FIM mode) or by electrons (in the FEM mode), and a supply chamber for the high purity gaseous reactants (Messer O_2: 99.999 %, Messer H_2: 99.999 %). The temperature of the specimen nanotip was measured by a Ni/NiCr thermocouple spot-welded to its shank. Particular attention was paid to the control of the specimen temperature: keeping the temperature strictly constant is an essential prerequisite for studying isothermal oscillations in the low-pressure regime of the H_2 oxidation reaction. For this purpose, a novel temperature control system based on wireless data transmission was used, which can operate in a temperature range from 77 to 900 K (*1*).

The Rh nanotip used as a specimen was sharpened by electrochemical etching of a Rh wire (0.1 mm, Mateck, 99.99 %). A salt melt (4 parts K_2NO_3 and 1 part KCl) was used as electrolyte with the Rh specimen acting as anode at a DC voltage of up to 4 V. Fine shaping and cleaning of the tip apex surface was achieved by field evaporation at 77 K in UHV. For monitoring the shaping progress and for the postreaction control, the tip apex was imaged with atomic resolution using FIM with Ne^+ ions as imaging species. The FIM images during the tip shaping procedures and the FEM videos during the ongoing H_2 oxidation were recorded by a CCD camera (Hamamatsu C9300-201). For the proper balance between the acquisition speed and signal-to-noise ratio, a framerate of 50 fps was chosen for *in situ* video recording of the ongoing reaction.

S2: Proper Orthogonal Decomposition (POD)

In the present study, we used proper orthogonal decomposition (POD), also known as Karhunen-Loève decomposition or principal components analysis, to analyze the *in situ* recorded FEM video data of the catalytic H_2 oxidation on a Rh nanotip. Given a set of spatio-temporal data, the method yields orthogonal spatial basis functions ("POD modes") and their time-dependent amplitudes, as well as the weight of the relative contribution of each basis function to the signal (*2*). The basis is "optimal", providing the minimal mean square truncation error in comparison with any other basis (*3*). The POD reconstruction of a spatio-temporal signal $W(\boldsymbol{x}, t)$, where $\boldsymbol{x}$ is the position vector, consists of a constant contribution of a time-averaged signal $R(\boldsymbol{x})$ and a combination of time-independent modes (spatial basis functions) $b_n(\boldsymbol{x})$ with their time-dependent amplitudes (time coefficients) $a_n(t)$ (Eq. S1, Fig. S1a).

$$W(\boldsymbol{x}, t) = R(\boldsymbol{x}) + \sum_{n=1}^{\infty} a_n(t) \cdot b_n(\boldsymbol{x}) \qquad \text{(Eq. S1).}$$

Both the basis functions $b_n(\boldsymbol{x})$ and their amplitudes $a_n(t)$ are equally important, as the basis functions show coherent spatial structures and the amplitudes describe how each structure evolves in time.

First, the time-averaged signal $R(\boldsymbol{x})$ is subtracted, so that only the varying part of the signal $U(\boldsymbol{x}, t) = W(\boldsymbol{x}, t) - R(\boldsymbol{x})$ is analyzed (*4*). The basis functions $b_n(\boldsymbol{x})$ are then the eigenvectors of the correlation matrix *C*, with $C_{i,j} = \langle U(\boldsymbol{x}_i, t), U(\boldsymbol{x}_j, t) \rangle_t$ representing the two-points correlation functions, where $\boldsymbol{x}_i$ and $\boldsymbol{x}_j$ represent the image pixels and $\langle \, \rangle_t$ denotes the time average. All basis functions are calculated by solving the eigenvalue equation $C \cdot B(\boldsymbol{x}) = \Lambda \cdot B(\boldsymbol{x})$, where $B(\boldsymbol{x})$ is the basis matrix composed of all $b_n(\boldsymbol{x})$ and the vector Λ contains the corresponding eigenvalues λ_n. The amplitudes $a_n(t)$ are obtained by projecting the varying signal parts $U(\boldsymbol{x}, t)$ onto the basis $B(\boldsymbol{x})$.

Each spatial mode together with its amplitude captures a certain percentage of the signal, whereas the magnitude of the eigenvalue λ_n is the measure of the significance of the corresponding mode. In this sense, the spectrum of eigenvalues can be used to reduce the complexity and to separate the dominant spatial structures from the obscuring background processes. In the case of only a few dominant modes capturing most of the signal, together with their time-dependent amplitudes they provide a low-dimensional representation of the original (high-dimensional) data (*5*).

Figure S1b-d shows an example of the POD application to the FEM video recorded at 430 K: the time-averaged $R(\boldsymbol{x})$ contribution is shown in Fig. S1b and the first three spatial POD modes with their respective time-dependent amplitudes are presented in Fig. S1c. The corresponding spectrum of eigenvalues (weight distribution) is shown in Fig. S1d. In the present case, the first POD mode contributes with a relative weight of 96.5 %, while the second and third mode have significantly lower weights of 1.1 % and 0.6 %, respectively. Therefore, in the present case already the first spatial mode together with its time-dependent amplitude and the time-averaged image $R(\boldsymbol{x})$ can largely approximate the original FEM signal (*cf.* "original" video (Supporting Movie S1) and the "first mode" video (Supporting Movie S2)).

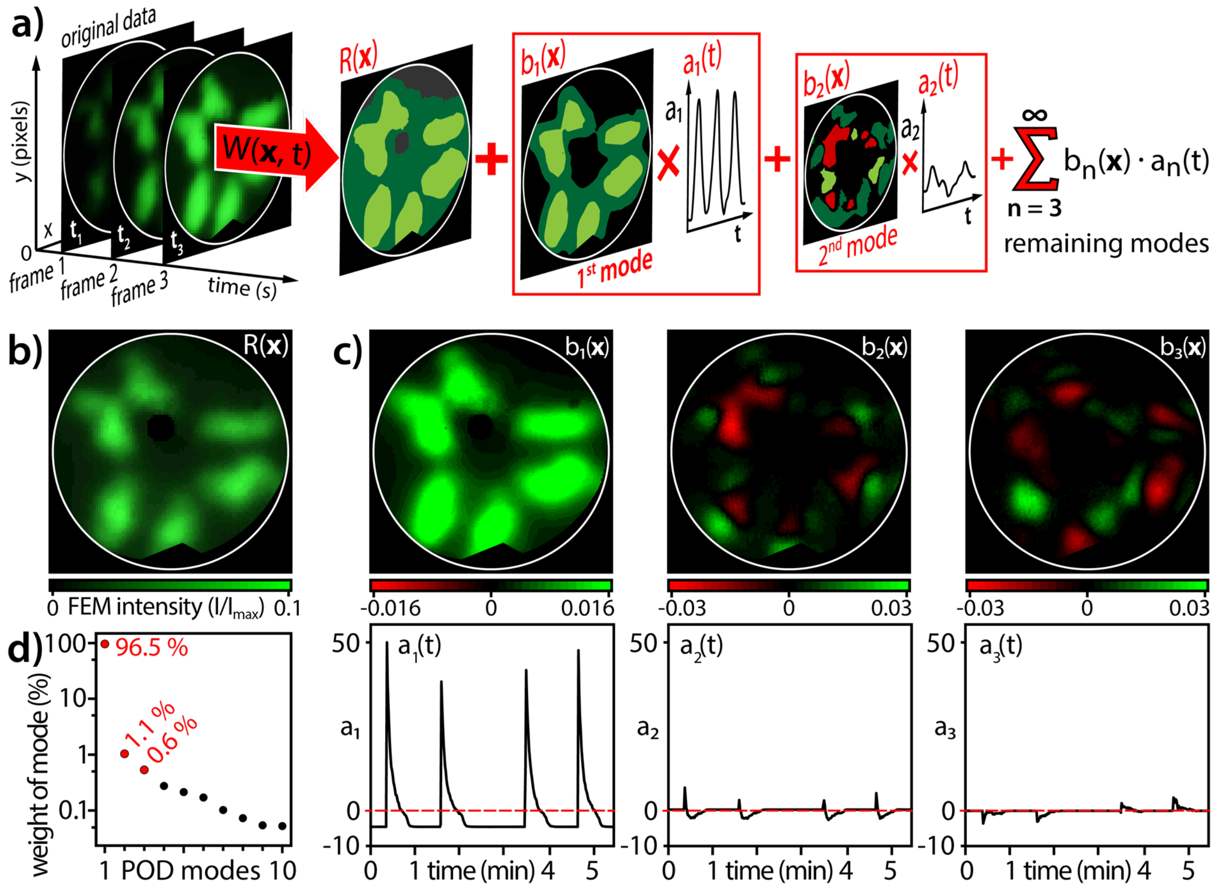

Figure S1. Proper orthogonal decomposition of FEM video data: a) original FEM video data (Supporting Movie S1) serve as spatio-temporal input signal $W(\boldsymbol{x}, t)$. The signal $W(\boldsymbol{x}, t)$ can be decomposed into a time-averaged image $R(\boldsymbol{x})$, spatial "modes" $b_n(\boldsymbol{x})$ and their respective time coefficients $a_n(t)$; b) constant image $R(\boldsymbol{x})$ calculated for the FEM video S1 recorded at 430 K. The FEM intensity scale is normalized to the maximum intensity I_{max} of the original FEM video; c) the first three POD modes and their respective time coefficients calculated from the FEM video S1 at 430 K; d) relative weight of the first ten POD modes (logarithmic scale).

S3: Transition Point Tracking (TPT)

The observed self-sustaining oscillations in H_2 oxidation on Rh (Supporting Movie S1) exhibit "blinking" character involving fast-spreading reaction fronts. The huge dynamic range of the FEM signal and the rapid spreading of the reaction fronts (relative to the acquisition speed of the video camera) makes the tracking of the propagation of reaction fronts and detection of the pacemaker locations difficult.

In order to overcome the difficulties, a novel method to track the rapid reaction fronts was developed, based on the fact that a reaction front is nothing else than a traveling continuum of kinetic transition points. Transition point tracking (TPT) extracts the local transition time as an inflection point from the time dependence of the local FEM image intensity for each pixel. Since the local FEM image intensity is related to the reaction rate (*6*), the spatial continuum of such inflection points at a given time provides a 2D transition map of momentary positions of the reaction front. The evolution of the transition map reflects the propagation of the reaction front. The algorithm for the construction of such a transition map is schematically explained in Fig. S2. The FEM video consists of a stack of frames recorded with a given framerate (Fig. S2a). The necessary balance between the acquisition speed and signal to noise ratio limits the framerate (50 fps in the present case). To improve the evaluation of the time dependent processes, a reconstruction of the *continuous* intensity progression from the recorded *discrete* data points for each pixel is desirable. The continuous intensity curves (Fig. S2b) reconstructed pixelwise allow a more precise assignment of the kinetic transition points in time. For this purpose, a sigmoidal time dependency of the activity during the kinetic transition is assumed. Initially, exponential growth of the local activity can be observed, resulting from the mechanism of the reaction (*7*): under suitable conditions, the initial catalytic formation of water molecules on the surface and their immediate desorption lead to more available adsorption sites for hydrogen, increasing the local reaction rate (*8, 9*). This autocatalytic behavior initiates the kinetic transition to the active state. The growth of activity, however, is eventually limited by the number of locally available adsorption sites, causing the activity to level-off locally. The combination of the above factors forms a particular shape of experimentally observed intensity curves in this and previous studies (*1, 10*). This justifies the assumption of a logistic growth, which is why the continuous intensity curves can best be reconstructed by applying a logistic fit to the local time-series (Fig. S2c). The inflection point of the fit, representing the local kinetic transition time τ_a, is then determined by computing the maximum of the first derivative (red curve in Fig. S2c). Due to the continuity of

the fit, the transition time τ_a can also fall between two frames, improving the effective time resolution.

At a given time, many local kinetic transitions may occur simultaneously at different surface locations. The times of transition at different locations are summarized in a transition map which also provides the evolution of kinetic transitions by using a color-coded time scale (Fig. S2f). Such a “raw” transition map still displays some spatial imperfections, since the fitted intensity curves are now continuous in time, but there are still small local intensity jumps from “pixel to pixel”, *i.e.,* from each continuous intensity curve of the particular pixel to the intensity curves of neighboring pixels. The physical reason of such jumps lies mainly in the diffusion- and reaction-induced surface density fluctuations accompanying any surface reaction (*11*). To counteract this effect, the “raw” transition maps were treated by a region-based smoothing algorithm (see Fig. S2d-f): the transition time of an exemplary pixel A is determined by taking the “raw” transition time values of surrounding pixels (within a distance r, black circle in Fig. S2f) into consideration. Of all the local “raw” transition times within this circle, a histogram is plotted (Fig. S2d). Fluctuation induced outliers are then removed by reducing the histogram to its main peak. From the remaining values within the circle, the new transition time (τ) is determined by weighted averaging (Eq. S2),

$$\tau = \frac{\sum_{i=1}^{N} \tau_{raw,i} \cdot \varepsilon_i \cdot w_i}{\sum_{i=1}^{N} \varepsilon_i \cdot w_i} \qquad \text{(Eq. S2)}$$

where *N* is the number of pixels within the circle and $\tau_{raw,i}$ is the “raw” local transition time. The parameter ε_i is 1 if the respective “raw” transition time is included in the main peak of the histogram and 0 if it is considered an outlier (Fig. S2d). The weight w_i of the value for each surrounding pixel is defined using a Gauss relation based on its distance to the center pixel A (Fig. S2e). Additionally, the standard deviation for the width of the gauss relation is determined for each circle separately: regions with a broader time distribution (90 % quantile – 10 % quantile) have narrower Gauss functions (smaller standard deviation) resulting in the weight distribution being shifted towards the center pixel. This procedure ensures a high accuracy of the generated transition map (Fig. S2g) for the determination of nucleation centers and regions of fast front propagation.

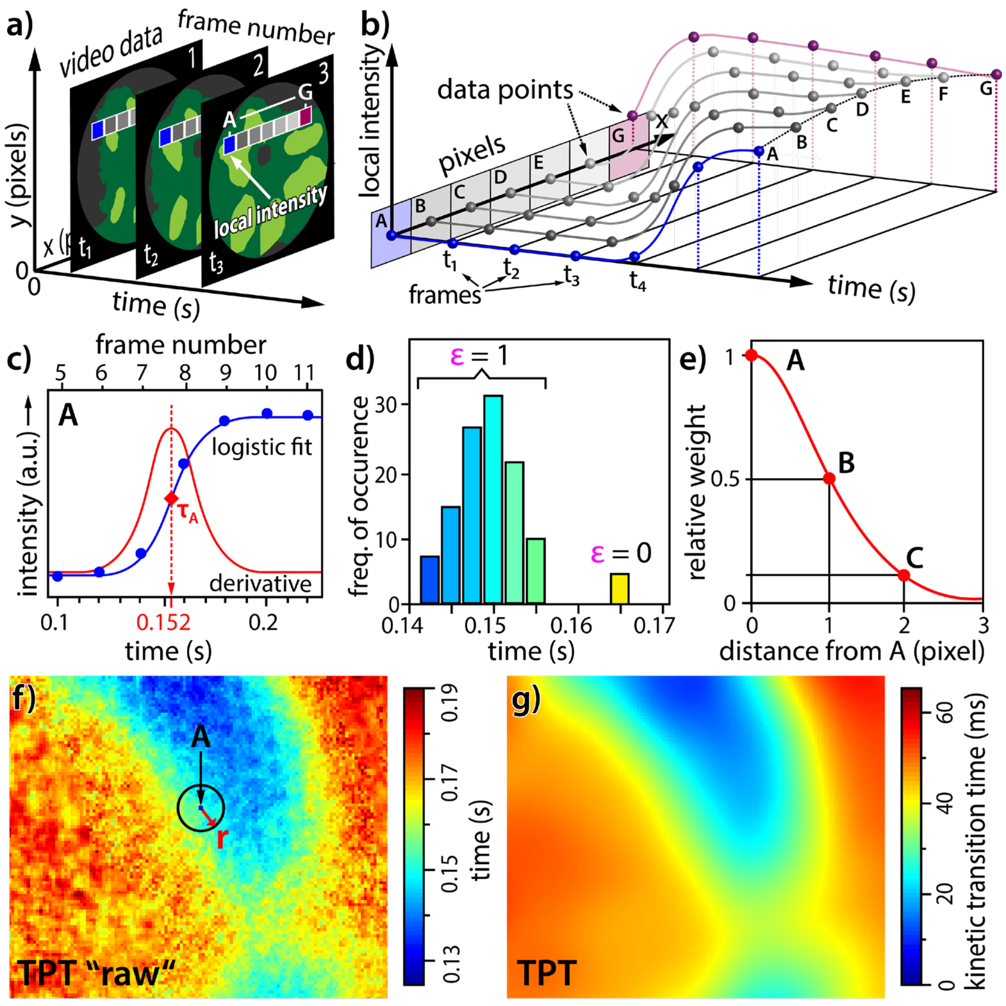

Figure S2. Transition Point Tracking (TPT): a) FEM video data as a stack or arrayed local time series of individual pixel intensities $I_i(t_n)$; b) conversion of discrete pixel intensities $I_i(t_n)$ into continuous "true time" curves $I_i(t)$; c) The local kinetic transition time point (red diamond) for the location of the exemplary pixel A is determined by the maximum of the first derivative (red curve) of the "true time" $I_A(t)$ dependence reconstructed by a logistic fit (blue curve) of the discrete data points (blue circles); d) occurrence histogram of the "raw" local transition times for the pixels neighboring to A (inside of the black circle in (f)); e) the weight of each included pixel decreases with its distance to A *via* a Gauss relation; f) "raw" transition map resulting directly from the pixelwise reconstruction of the local intensity curves; g) the same, but after applying the region-based smoothing algorithm. The time scale is zeroed to the earliest local transition.

S4: Micro-kinetic model simulations

The model simulations were performed using a mean-field microkinetic model based on the Langmuir-Hinshelwood mechanism that is well established for H_2 oxidation on Rh surfaces. To describe the rate oscillations, the periodic formation and depletion of subsurface oxygen is considered as the feedback mechanism (*12, 13*).

The full oscillating cycle can be described by the following main steps: (1) oxygen adsorbs dissociatively *via* a molecular precursor on the Rh surface (Eq. S3 and S4); (2) adsorbed oxygen diffuses from the surface to empty subsurface sites, forming subsurface oxygen (Eq. S5). The presence of subsurface oxygen hinders the adsorption of further oxygen by significantly reducing its effective sticking coefficient; (3) hydrogen can still adsorb on available neighboring empty surface sites (Eq. S6); (4) From the co-adsorbed reactants, H_2O is formed by reaction (Eq. S7, OH intermediates are not considered in the model). As a result of facile water formation, the surface is emptied of adsorbed oxygen, causing subsurface oxygen atoms to diffuse to the surface. In turn, the depletion of the subsurface oxygen reservoir recovers the high sticking coefficient of oxygen. Once more, oxygen adsorbs predominantly, thereby closing the oscillation cycle. The above steps of the cycle can be formulated as five reaction equations (Eq. S3-7),

$$O_2 \leftrightarrow O_{2,pre} \quad (\text{Eq. S3})$$

$$O_{2,pre} + 2\ * \leftrightarrow 2\ O_{ads} \quad (\text{Eq. S4})$$

$$O_{ads} + *_{sub} \leftrightarrow O_{sub} + * \quad (\text{Eq. S5})$$

$$H_2 + 2\ * \leftrightarrow 2\ H_{ads} \quad (\text{Eq. S6})$$

$$O_{ads} + 2\ H_{ads} \leftrightarrow\ H_2O + 3\ * \quad (\text{Eq. S7})$$

where an empty surface site is represented by $*$. The described cycle assumes immediate desorption of the reaction product water. This assumption is reasonable because water is known to desorb from Rh surfaces at around 300 K (*14*), whereas our microkinetic simulations are performed for temperatures above 430 K. The applied model is a modified version of the model used by McEwen *et al.* to simulate field-induced oscillations in H_2 oxidation (*15, 16*). In the present calculation, the model was adapted to field-free reaction conditions (see Supporting Text S5). Accordingly, the effective dipolar dependences of processes assuming the field-dependence of the

binding energy of reactants could be neglected. This modified approach has already been applied in previous studies of the catalytic hydrogen oxidation reaction on Rh (*12, 13*). Within the present model, the following kinetic equations describe the coverages for oxygen, subsurface oxygen and hydrogen (Eq. S8-10):

$$\frac{\mathrm{d}\theta_{\mathrm{O}}}{\mathrm{d}t} = \frac{2}{1+K\theta_*{}^2}\left(k_{\mathrm{a}}^{\mathrm{O}} K p_{\mathrm{O}_2}\theta_*{}^2 - k_{\mathrm{d}}^{\mathrm{O}}\theta_{\mathrm{O}}{}^2\right) - k_{\mathrm{ox}}\theta_{\mathrm{O}}(1-\theta_{\mathrm{s}}) + k_{\mathrm{red}}\theta_{\mathrm{s}}\theta_* - k_{\mathrm{r}}\theta_{\mathrm{H}}\theta_{\mathrm{O}} \quad (\mathrm{Eq.\,S8})$$

$$\frac{\mathrm{d}\theta_{\mathrm{s}}}{\mathrm{d}t} = k_{\mathrm{ox}}\theta_{\mathrm{O}}(1-\theta_{\mathrm{s}}) - k_{\mathrm{red}}\theta_{\mathrm{s}}\theta_* \quad (\mathrm{Eq.\,S9})$$

$$\frac{\mathrm{d}\theta_{\mathrm{H}}}{\mathrm{d}t} = 2k_{\mathrm{a}}^{\mathrm{H}} p_{\mathrm{H}_2}\theta_*{}^2 - 2k_{\mathrm{d}}^{\mathrm{H}}\theta_{\mathrm{H}}^2 - 2k_{\mathrm{r}}\theta_{\mathrm{H}}\theta_{\mathrm{O}} \quad (\mathrm{Eq.\,S10})$$

The empty sites are given by $\theta_* = 1-\theta_{\mathrm{H}}-\theta_{\mathrm{O}}$ and the rate constants are given by (Eq. S11-18):

$$k_{\mathrm{a}}^{\mathrm{H}} = S_0^{\mathrm{H}}\, a_{\mathrm{s}}/\sqrt{2\pi m_{\mathrm{H}_2} k_{\mathrm{B}} T} \quad (\mathrm{Eq.\,S11})$$

$$k_{\mathrm{a}}^{\mathrm{O}} = S_0^{\mathrm{O}}\, a_{\mathrm{s}}/\sqrt{2\pi m_{\mathrm{O}_2} k_{\mathrm{B}} T} \quad (\mathrm{Eq.\,S12})$$

$$k_{\mathrm{d}}^{\mathrm{H}} = k_{\mathrm{d0}}^{\mathrm{O}} e^{-\beta E_{\mathrm{d}}^{\mathrm{H}}} \quad (\mathrm{Eq.\,S13})$$

$$K = K_0 e^{-\beta(E_{\mathrm{K}} + A_{\mathrm{K}}^{\mathrm{O}}\,\theta_{\mathrm{O}} + A_{\mathrm{K}}^{\mathrm{S}}\theta_{\mathrm{s}})} \quad (\mathrm{Eq.\,S14})$$

$$k_{\mathrm{d}}^{\mathrm{O}} = k_{\mathrm{d0}}^{\mathrm{O}} e^{-\beta(E_{\mathrm{d}}^{\mathrm{O}} + A_{\mathrm{d}}^{\mathrm{O}}\,\theta_{\mathrm{O}} + B_{\mathrm{d}}^{\mathrm{O}}\,\theta_{\mathrm{O}}{}^2)} \quad (\mathrm{Eq.\,S15})$$

$$k_{\mathrm{ox}} = k_{\mathrm{ox}}^{0} e^{-\beta E_{\mathrm{ox}}} \quad (\mathrm{Eq.\,S16})$$

$$k_{\mathrm{red}} = k_{\mathrm{red}}^{0} e^{-\beta(E_{\mathrm{red}} + A_{\mathrm{red}}^{\mathrm{S}}\theta_{\mathrm{s}})} \quad (\mathrm{Eq.\,S17})$$

$$k_{\mathrm{r}} = k_{\mathrm{r}}^{0} e^{-\beta(E_{\mathrm{r}} + A_{\mathrm{r}}^{\mathrm{H}}\theta_{\mathrm{H}} + A_{\mathrm{r}}^{\mathrm{O}}\theta_{\mathrm{O}})} \quad (\mathrm{Eq.\,S18})$$

The sticking probabilities for the reactants at the start of the calculations are given by S_0^{H} and S_0^{O}, respectively, while m_{H_2} and m_{O_2} represent the molecular masses. The area of a surface site (a_s) is set to 10 Å^2 and $\beta = 1/k_{\mathrm{B}}T$.

Table S1 contains the values of the kinetic parameters used for our calculations presented in Fig. 6. These values match those previously determined for Rh(110) except the activation energy of oxide formation E_{ox} and the coverage dependence of sub-surface oxygen on surface-subsurface reduction ($A_{\mathrm{red}}^{\mathrm{s}}$). The best fit to our experimental data was obtained using the Rh(110) value-set selected from value-sets for low-Miller-index surfaces tabulated in Ref. (*15*). This indicates that the properties of the pacemaker regions governing the oscillations (in our case Rh{973} at 430 K) can be approximated with reasonable accuracy using the most corrugated Rh low-index-surface. Unfortunately, corresponding value-sets for high-Miller-index Rh surfaces are not available. Performing the simulations, E_{ox} was varied until oscillating behavior was observed and E_{red} was calculated accordingly using the scaling relation $E_{\mathrm{red}} = 0.293 + 0.776\, E_{\mathrm{ox}}$. As we investigate highly stepped and kinked Rh nanofacets (particularly the pacemaker regions, Fig. 6a), it is reasonable to expect even lower oxidation barriers, thus $A_{\mathrm{red}}^{\mathrm{s}}$ was reduced from 0.3 eV used in Ref. (*15*) to 0.27 eV, for obtaining a correct temperature dependency of the oscillating frequency. Keeping other parameters constant, one obtains a continuous oscillation regime in the experimentally investigated temperature range of 430 K to 450 K, limited at a minimal E_{ox} of 1.037 eV (corresponding to the highest oscillation frequencies) and a maximum E_{ox} of 1.121 eV (corresponding to the lowest oscillation frequencies) for hydrogen and oxygen pressures of 2.3×10^{-6} and 1.6×10^{-6} mbar, respectively. At these hydrogen and oxygen pressures, the frequencies obtained from the experiments are best fitted using an E_{ox} value of 1.048 eV, as can be seen in Fig. 6b. This value of 1.048 eV is significantly lower than the value of 1.68 eV used in Ref. (*15*) for Rh(111). When set in relation to our previous studies for stepped Rh surfaces of a polycrystalline foil (*12, 13*), the E_{ox} range for oscillation conditions for the present nanotip lies even lower than the therein estimated values of 1.111 eV and 1.134 eV for the foil, and 1.085 eV, 1.100 eV and 1.108 eV for the curved Rh crystal (*17*). This is in accord with the generally higher corrugation (and thereby lower activation energy for subsurface formation) of nanofacets on the highly curved Rh tip apex with respect to corresponding macroscopic planar surfaces of single crystals and mesoscopic domains of polycrystalline foils.

Table S1. Parameters used in our micro-kinetic simulations. The energies for surface oxidation and reduction are given in the text. Energies are given in eV, the area of a surface site (a_s) in Å^2 and rate constants in 1/s.

a_s	10
E_d^H	0.64
S_0^O	0.95
A_K^s	0.075
E_d^O	3.2
A_d^O	-0.5
B_d^O	-0.7
E_r	0.79
S_0^H	0.3
k_{d0}^h	3.0 x 10^{10}
K_0	0.2525
E_k	-0.178
A_K^O	0.158
k_{ox}^0	5.0 x 10^{11}
k_{red}^0	1.85 x 10^{13}
A_{red}^s	0.27
k_{d0}^O	6.0 x 10^{13}
k_r	7.0 x 10^{12}
A_r^H	-0.27
A_r^O	-0.145

S5: The role of the electric field

Discussing the FEM results, the possible effect of the applied electrostatic field required for field emission imaging should be mentioned. The field effects that can occur when a high electrostatic field is applied to a metal surface are related to the field induced redistribution of the electron density near the surface. Although it is difficult to measure the spatial electron density distribution under high field conditions, some evaluation possibilities are provided by field ion microscopy (FIM (*18*)), scanning tunneling microscopy (STM (*19*)) and field ion appearance energy spectroscopy (FIAES (*20, 21*)). Using these techniques, local field variations can be estimated, which are unambiguously related to the electron density *via* the Poisson equation.

It is also possible to calculate the field-modified surface electron densities and the resulting field distributions (*22, 23*). For close-packed surfaces of catalytically important precious metals (Pt, Rh, Pd), the jellium model based calculations appeared to be successful (*24, 25*). An example of such a field induced redistribution is shown in Fig. S3, where the field-free and field-modified electron density distributions calculated within the jellium model using the functional integration method are presented (*26*). This method allows correct consideration of the exchange-correlation effects and makes a proper account of the field-effect for broad field ranges possible without using the perturbation theory.

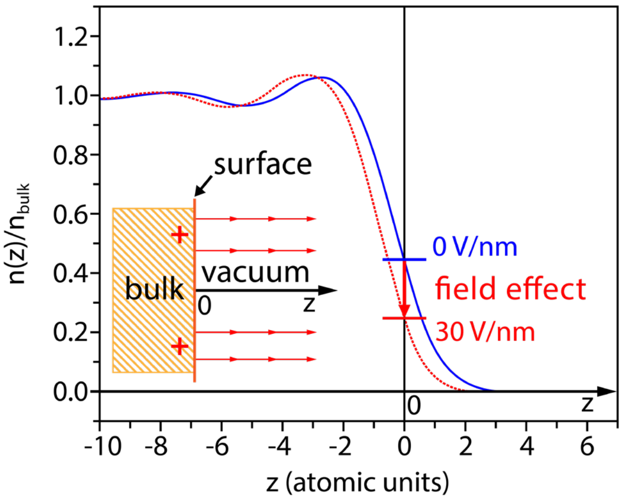

Fig. S3. Electron density distributions near a flat metal jellium with r_s = 3.0 a. u. (≈ 1.6 Å). The blue solid line corresponds to zero field, the red dashed line to an external field of 30 V/nm. The field modified electron density is remarkably shifted towards the bulk, *i.e.,* the surface electron density (at z = 0) is effectively lowered. The electrostatic field is parallel to z-direction, *i.e.*, the surface is positively charged. Based on Ref. (*26*).

The field modified electron density distribution may change the binding energy of adsorbed reactants and thus the kinetics of the catalyzed surface reaction (*27*). For example, the influence of a high electrostatic field on CO oxidation on Pt has been detected (*28*), which is comparable in scale to the influence caused by the co-adsorption of electropositive adatoms such as alkali or rare earth metals (*29*). The field strength values play, of course, an important role as well: any measurable field effects on the adsorption energy of typical reactants such as oxygen or hydrogen are not expected for field values less than 5 V/nm.

Everything mentioned so far concerns an externally applied "positive" field, in the sense that the metal surface is positively charged, *i.e.,* the field strength vector is directed away from the surface, as drawn schematically in Fig. S3. Experimentally, this corresponds to the FIM configuration with a nanotip acting as a sample being positively charged. Consequently, field effects on the adsorption and catalytic reaction (*e.g.* CO oxidation on Pt) were observed in FIM experiments (*28*).

In turn, in an FEM, where the sample is always negatively charged, on a first glance an opposite field effect could be expected. However, a corresponding shift of electron density in the opposite direction (towards vacuum) caused by a negatively charged surface is not possible: the onset of field emission of electrons (< 4 V/nm for most metals) is reached way before the field strength reaches values at which observable electron density modifications can occur. A previous CO oxidation study on Pt using a pulsed high-voltage supply with varying duty pulses provided direct experimental evidence of this assessment and justifies considering the present FEM studies as quasi field-free (*28*). In that respect, the present FEM studies differ from the previous FIM studies of H_2 oxidation (*30*) where a field effect was observed. Moreover, the high "positive" electric field in FIM may even lead to field-induced reaction-rate oscillations under these reaction conditions (*16*). In contrast, oscillations observed in the present study are caused solely by non-linear dynamics of the reaction process coupled with the subsurface oxygen-based feed-back mechanism, as recently detected on the µm-scale (*12, 13*).

References

(*1*) Winkler, P.; Zeininger, J.; Raab, M.; Rupprechter, G.; Suchorski. Y. A novel wireless sample temperature control system for field ion, field electron, and atom probe techniques. *Rev. Sci. Instrum.* **2020,** *91,* 013705.

(*2*) Weiss, J. A Tutorial on the proper orthogonal decomposition. In *AIAA Aviation 2019 Forum, Dallas, Texas, United States, June 17-21,* 2019, American Institute of Aeronautics and Astronautics, AIAA 2019-3333*,* 1-21.

(*3*) Lahme, B.; Miranda R. Karhunen-Loève decomposition in the presence of symmetry. *IEEE T. Image Process*. **1999,** *8,* 1183-1190.

(*4*) Graham, M. D.; Kevrekidis, I. G.; Hudson, J. L.; Veser, G.; Krischer, K.; Imbihl, R. Dynamics of concentration patterns of the NO + CO reaction on Pt: Analysis with the Karhunen-Loève decomposition. *Chaos, Soliton. Fract.* **1995,** *5,* 1817-1831.

(*5*) Gutierrez-Castillo, P.; Thomases, B. Proper orthogonal decomposition (POD) of the flow dynamics for a viscoelastic fluid in a four-roll mill geometry at the stokes limit. *J. Non-Newton. Fluid* **2019,** *264*, 48-61.

(*6*) Suchorski, Y.; Rupprechter, G. Local reaction kinetics by imaging. *Surf. Sci.* **2016,** *643,* 52-58.

(*7*) Zum Mallen, M. P.; Williams, W. R.; Schmidt, L. D. Steps in hydrogen oxidation on rhodium: hydroxyl desorption at high temperatures. *J. Phys. Chem.* **1993,** *97*, 625-632.

(*8*) Africh, C.; Lin, H.; Corso, M.; Esch, F.; Rosei, R.; Hofer, W. A.; Comelli, G. Water production reaction on Rh (110). *J. Am. Chem. Soc.* **2005,** *127,* 11454-11459.

(*9*) Africh, C.; Comelli, G. Scanning tunnelling microscopy investigations of simple surface reactions on Rh (110). *J. Phys.-Condens. Mat.* **2006,** *18,* R387.

(*10*) Vogel, D.; Spiel, C.; Suchorski, Y.; Trinchero, A.; Schlögl, R.; Grönbeck, H.; Rupprechter, G. Local catalytic ignition during CO oxidation on low-index Pt and Pd surfaces: a combined PEEM, MS, and DFT study. *Angew. Chem. Int. Edit.* **2012,** *51*, 10041-10044.

(*11*) Suchorski, Y.; Beben, J.; Imbihl, R.; James, E. W.; Liu, D. J.; Evans, J. W. Fluctuations and critical phenomena in catalytic CO oxidation on nanoscale Pt facets. *Phys. Rev. B*, **2001,** *63,* 165417.

(*12*) Suchorski, Y.; Datler, M.; Bespalov, I.; Zeininger, J.; Stöger-Pollach, M.; Bernardi, J.; Grönbeck, H.; Rupprechter G. Visualizing catalyst heterogeneity by a multifrequential oscillating reaction. *Nature Communications* **2018,** *600,* 1-6.

(*13*) Suchorski, Y.; Datler, M.; Bespalov, I.; Zeininger, J.; Stöger-Pollach, M.; Bernardi, J.; Grönbeck, H.; Rupprechter G. Surface-structure libraries: multifrequential oscillations in catalytic hydrogen oxidation on rhodium. *J. Phys. Chem. C,* **2019,** *123,* 4217-4227.

(*14*) Gregoratti, L.; Baraldi, A.; Dhanak, V. R.; Comelli, G.; Kiskinova, M.; Rosei, R. Structural effects on water formation from coadsorbed H + O on Rh(100). *Surf. Sci.* **1995,** *340,* 205-214.

(*15*) McEwen, J.-S.; Gaspard, P.; Visart de Bocarmé, T.; Kruse, N. Electric field induced oscillations in the catalytic water production on rhodium: a theoretical analysis. *Surf. Sci.* **2010,** *604,* 1353-1368.

(*16*) McEwen, J.-S.; Gaspard, P.; Visart de Bocarmé, T.; Kruse, N. Oscillations and bistability in the catalytic formation of water on rhodium in high electric fields. *J. Phys. Chem. C* **2009,** *113,* 17045-17058.

(*17*) Suchorski, Y.; Zeininger, J.; Buhr, S.; Raab, M.; Stöger-Pollach, M.; Bernardi, J.; Grönbeck, H.; Rupprechter G. Resolving multifrequential oscillations and nanoscale interfacet communication in single particle catalysis. *Science,* **2021,** *372,* 1314-1318.

(*18*) Suchorski, Y.; Schmidt, W. A.; Block, J. H. Enhanced local electric fields in field ionization at steps of clean and Au-covered Rh (111). *Appl. Surf. Sci.* **1993,** *67,* 124-127.

(*19*) Miskovsky, N. M.; Tsong, T. T. Field evaporation of gold in single-and double-electrode systems. *Phys. Rev. B* **1992,** *46,* 2640.

(*20*) Suchorski, Y.; Schmidt, W. A.; Ernst, N.; Block, J. H.; Kreuzer, H. J. Electrostatic fields above individual atoms. *Prog. Surf. Sci.* **1995,** *48,* 121-134.

(*21*) Schmidt, W. A.; Ernst, N.; Suchorski, Y. Local electric fields at individual atomic surface sites: field ion appearance energy measurements. *Appl. Surf. Sci.* **1993,** *67,* 101-110.

(*22*) Aers, G. C.; Inglesfield, J. E. Electric field and Ag (001) surface electronic structure. *Surf. Sci.* **1989,** *217,* 367-383.

(*23*) Kreuzer, H. J.; Wang, L. C.; Lang, N. D. Self-consistent calculation of atomic adsorption on metals in high electric fields. *Phys. Rev. B* **1992,** *45,* 12050.

(*24*) Gies, P.; Gerhardts, R. R. Self-consistent calculation of electron-density profiles at strongly charged jellium surfaces. *Phys. Rev. B* **1986,** *33,* 982.

(*25*) Gohda, Y.; Nakamura, Y.; Watanabe, K.; Watanabe, S. Self-consistent density functional calculation of field emission currents from metals. *Phys. Rev. Lett.* **2000,** *85,* 1750-1753.

(*26*) Kostrobiy, P. P.; Markovych, B. M.; Suchorski, Y. Revisiting local electric fields on close-packed metal surfaces: theory versus experiments. *Sol. St. Phen.* **2007,** *128,* 219-224.

(*27*) Sieben, B.; Suchorski, Y.; Bozdech, G.; Ernst, N. Interaction of CO and O_2 with Pt studied by field ion appearance energy spectroscopy. *Z. Phys. Chem.* **1997,** *202,* 103-115.

(*28*) Suchorski, Y.; Imbihl, R.; Medvedev, V. K. Compatibility of field emitter studies of oscillating surface reactions with single crystal measurements: catalytic CO oxidation on Pt. *Surf. Sci.* **1998,** *401,* 392-399.

(*29*) Kiskinowa, M. P. Poisoning and promoting in catalysis based on surface science concepts and experiments. In *Studies in surface science and catalysis;* Delmon, B.; Yates, J. T., Eds.; Elsevier, Amsterdam, 1992.

(*30*) McEwen, J. S.; Ros, A. G. C.; Gaspard, P.; Visart de Bocarmé, T.; Kruse, N. Non-equilibrium surface pattern formation during catalytic reactions with nanoscale resolution: investigations of the electric field influence. *Catal. Today.* **2010,** *154,* 75-84.
